# Indocyanine green angiographic findings in a patient with AIDS and disseminated sporotrichosis

**DOI:** 10.5935/0004-2749.202200102

**Published:** 2022

**Authors:** Ana Luiza Biancardi, Raíssa Lima de Moraes, André Luiz Land Curi

**Affiliations:** 1 Laboratory of Infectious Ophthalmology, Instituto Nacional de Infectologia, Fundação Oswaldo Cruz, Rio de Janeiro, RJ, Brazil; 2 Instituto Nacional de Infectologia, Fundação Oswaldo Cruz, Rio de Janeiro, RJ, Brazil

To the editor,

Sporotrichosis is a subcutaneous infection caused by dimorphic fungi of the *Sprorothrix* complex, which is endemic in Latin America, South Africa, India, and Japan^(1)^. In Rio de Janeiro, Brazil, a rising number of cases associated with contact with cats has been reported since 1998^(2)^.

Adnexa and conjunctival sporotrichosis occurs by direct inoculation, while intraocular involvement is caused by hematogenous dissemination in immunosuppressed patients with disseminated sporotrichosis^(1,3)^.

This report describes a patient with acquired immunodeficiency syndrome (AIDS) in which a diagnosis of disseminated sporotrichosis was suspected after ophthalmic assessment revealing multifocal choroiditis on indocyanine green angiography.

A 31-year-old male patient presented with respiratory symptoms indicating pneumocystosis, leading to the diagnosis of AIDS. His CD4 count was 9 cells/m^3^, and antiretroviral therapy with tenofovir, lamivudine, and efavirenz was prescribed. Two weeks later, he presented with pain and low visual acuity in his left eye (OS). Also, multiple ulcerated skin lesions with erythematous edges at noncontiguous sites developed, and he had nasal mucosa lesions in the septum, leading to bloody secretion and detachment of crusts.

The ophthalmic assessment revealed visual acuity of 20/20 in the right eye (OD) and light perception in the OS. OS examination showed perforation and iris tamponade. Biomicroscopy and intraocular pressure were unremarkable in the OD. The fundoscopic examination was impossible in the OS, and there was multifocal choroiditis in the OD. Fundus photography showed multiple yellowish rounded lesions in the OD. Fluorescein angiography revealed multiple hypofluorescent lesions in the early phase and hyperfluorescent lesions in the late phase. Indocyanine green angiography (ICG) revealed rounded well-defined hypofluorescent lesions in the posterior pole and mid-periphery, indicating multifocal choroiditis (Figure 1).

**Figure 1:**
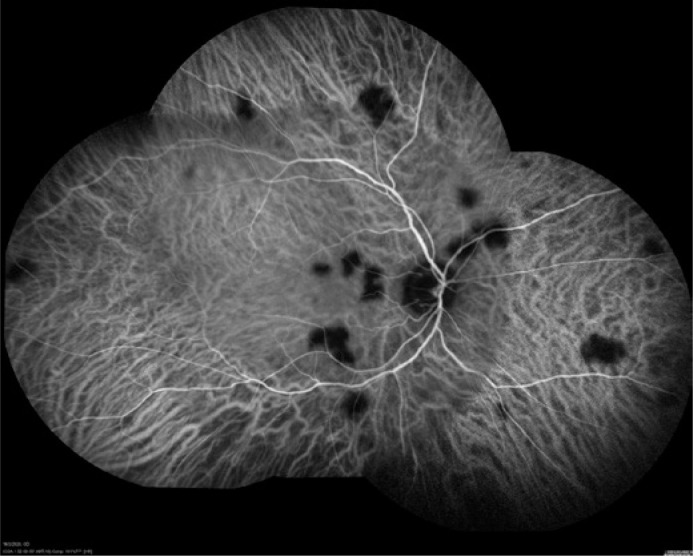
Indocyanine green angiography (ICG) of the right eye: Rounded well-defined hypofluorescent lesions in the posterior pole and mid-periphery, indicating multifocal choroiditis.

The results of the ophthalmic evaluation increased the possibility of disseminated sporotrichosis diagnosis. A skin biopsy was performed, and the culture was positive for *Sporothrix schenckii*. After the diagnosis of sporotrichosis, the patient reported a cat bite on his finger. Therefore, the patient was treated with intravenous amphotericin B, achieving full choroiditis recovery (Figure 2).

**Figure 2:**
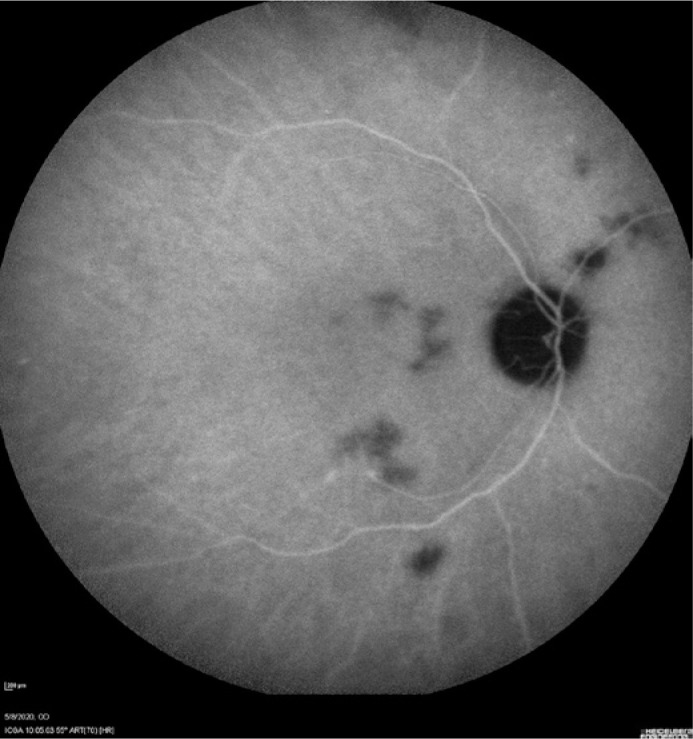
Improvement of these lesions after treatment with amphotericin B.

Infectious choroiditis has various manifestations that allow clinical suspicion of the pathogen. Worldwide, tu berculosis is a very common cause of infectious choroiditis occurring in immunocompetent and immunocompromised patients. Since it is difficult to isolate an infectious agent from the eye, choroiditis is usually a presumed diagnosis. Thus, the appearance, localization, and the number of choroidal lesions are features resulting in the suspicion of an infectious agent. Therefore, the association with clinical signs and symptoms is the benchmark for the diagnosis^(4)^.

Choroiditis is a rare manifestation of *Sporothrix spp* infection. It was described as a condition associated with severe immunosuppression in patients with HIV and disseminated sporotrichosis^(3)^.

In this case report, the diagnosis of pneumocystis choroiditis was suspected once pulmonary pneumocystosis was identified as the first opportunistic manifestation. However, the clinical features of pneumocystis choroiditis are different. Classically, this disorder presents with demarcated lesions located in the posterior pole, and it is usually associated with disseminated systemic pneumocystosis in patients with severe immunosuppression^(4)^. Thus, the treatment for this infection did not improve multifocal choroiditis.

The diagnosis of disseminated sporotrichosis with multifocal choroiditis was considered since the patient presented with multifocal choroiditis and disseminated skin lesions.

ICG is a cyanine dye that has ideal infrared frequencies to penetrate the retinal layers, allowing ICG angiography to image deeper circulation patterns than fluorescein angiography. Its importance is related to the detection of subclinical choroidal lesions and their follow-up^(5)^. In this case report, ICG angiography revealed more lesions than was observed during fundus examination, and it was useful to assess choroiditis recovery. In conclusion, ICG the exam showed a different pattern of choroiditis related to sporotrichosis from those previously described.

## References

[1] Arinelli A, Aleixo ALQC, Freitas DFS, do Vakke ACF, Almeida-Paes R, Gutierrez-Galhardo MC (2020). Ocular sporotrichosis: 26 cases with bulbar involvement in a
hyperendemic area of zoonotic transmission. Ocular Immunol Inflamm.

[2] Schubach A, Barros MB, Wanke B. (2008). Epidemic sporotrichosis. Curr Opin Infect Dis.

[3] Biancardi AL, Freitas DF, Valviesse VR, Andrade HB, de Oliveira MM, do Valle AC (2017). Multifocal choroiditis in disseminated sporotrichosis in patients
with HIV/AIDS. Retin Cases Brief Rep.

[4] Vrabec TR. (2004). Posterior segment manifestations of HIV/AIDS. Surv Ophthalmol.

[5] Agrawal RV, Biswas J, Gunasekaran D. (2013). Indocyanine green angiography in posterior
uveitis. Indian J Ophthalmol.

